# Publisher Correction: Role of outer surface probes for regulating ion gating of nanochannels

**DOI:** 10.1038/s41467-018-03030-4

**Published:** 2018-02-08

**Authors:** Xinchun Li, Tianyou Zhai, Pengcheng Gao, Hongli Cheng, Ruizuo Hou, Xiaoding Lou, Fan Xia

**Affiliations:** 1 State Key Laboratory of Material Processing and Die & Mould Technology, School of Material Sciences and Engineering, Hubei Key Laboratory of Bioinorganic Chemistry & Materia Medica, School of Chemistry and Chemical Engineering, Huazhong University of Science and Technology (HUST), 430074 Wuhan, China; 2 Pharmaceutical Analysis Division, School of Pharmacy, Guangxi Medical University, 530021 Nanning, China; 3 Faculty of Materials Science and Chemistry, China University of Geosciences, 430074 Wuhan, China

Correction to: *Nature Communications* 10.1038/s41467-017-02447-7, published online 03 January 2018

The original version of this Article contained an error in Fig. 3. The scale bars in Figs 3c and d were incorrectly labelled as 50 μA. In the correct version, the scale bars are labelled as 0.5 μA. This has now been corrected in both the PDF and HTML versions of the Article.

