# Supplementary material for: Role of outer surface probes for regulating ion gating of nanochannels
Source: Nat Commun. 2018 Jan 3;9:40. doi: 10.1038/s41467-017-02447-7 (PMC5752670; doi:10.1038/s41467-017-02447-7)
Supplement: Supplementary file 1 — Supplementary Information [file 41467_2017_2447_MOESM1_ESM.pdf]

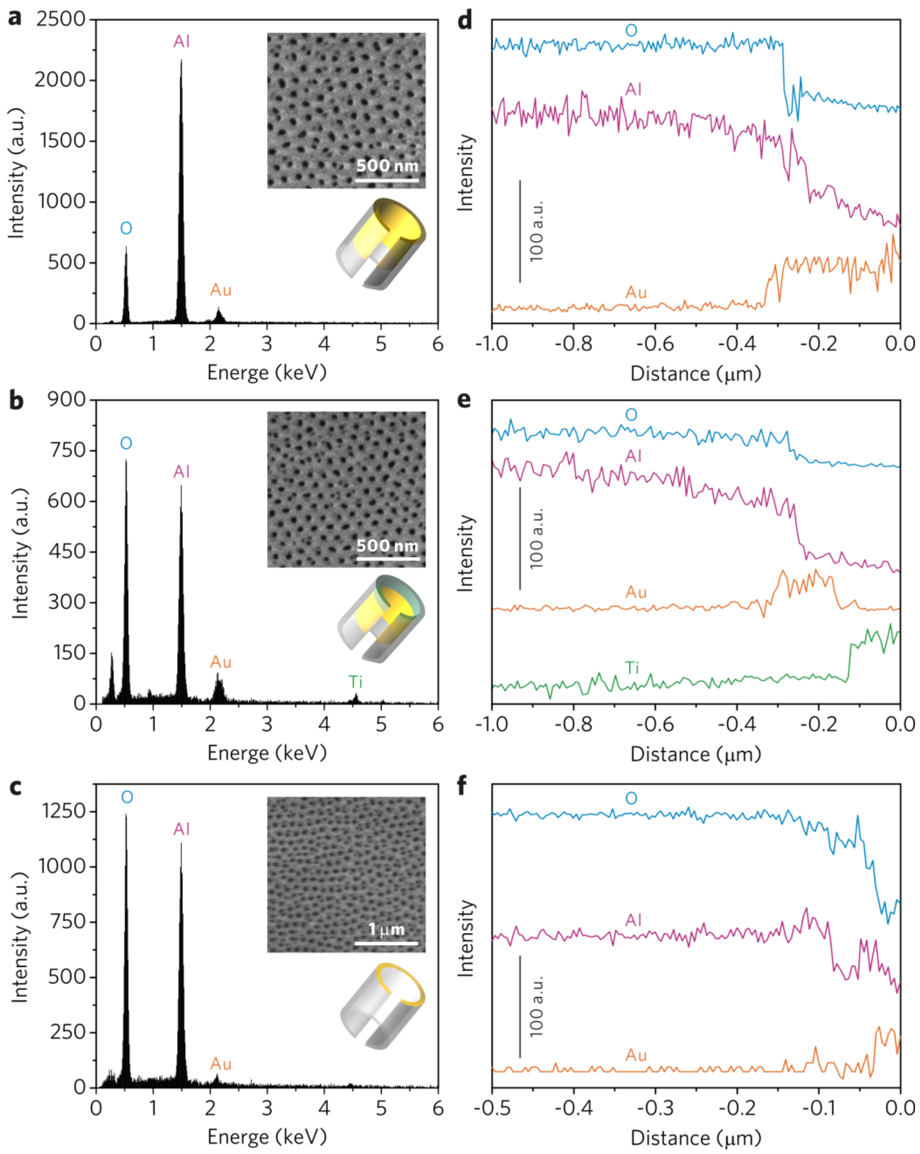

Supplementary Figure 1. (a-c) EDS data obtained from surface scan of 10 nm Au (a), 10 nm Au + 5 nm Ti (b), and 3 nm Au (c) decorated AAO nanochannels. Inset: SEM morphology and schematic description of the metal deposited AAO nanochannels. **(d-f)** The corresponding EDS results using linear scan mode to reveal the distribution of the selected elements in the nanochannels.

a

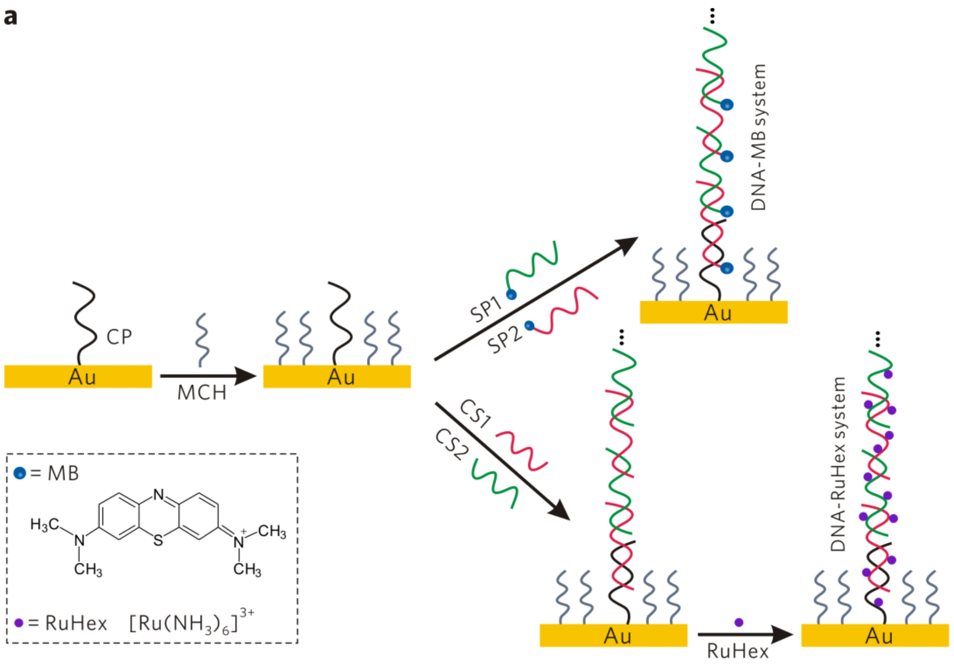

b

MB-DNA system	Sequence (traditional sandwich)
CP	5′-SH-$(CH_2)_6$-CGGC ACC TGG GGG AGT ATT GCG GAG GAA GGT GCCG-3′
SP1	5′-TACT CCC CCA GGT GCC GAC GGC ACC TTC CTC CGCA-MB-3′
SP2	5′-MB-CGGC ACC TGG GGG AGT AT-3′
MB-DNA system	Sequence (superstructure)
CP	5′-SH-$(CH_2)_6$-CGGC ACC TGG GGG AGT ATT GCG GAG GAA GGT GCCG-3′
SP1	5′-TACT CCC CCA GGT GCC GAC GGC ACC TTC CTC CGCA-MB-3′
SP2	5′-MB-CGGC ACC TGG GGG AGT ATT GCG GAG GAA GGT GCCG-3′
RuHex-DNA system	Sequence (superstructure)
CP	5′-SH-$(CH_2)_6$-CGGC ACC TGG GGG AGT ATT GCG GAG GAA GGT GCCG-3′
CS1	5′-TACT CCC CCA GGT GCC GAC GGC ACC TTC CTC CGCA-3′
CS2	5′-CGGC ACC TGG GGG AGT ATT GCG GAG GAA GGT GCCG-3′

Supplementary Figure 2. (a) Process for the assembly of DNA superstructures (DNA-SS) containing ATP binding aptamer at the nanochannels, by which molecular switch and ion gating systems can thereby be established. Note that electrolytic current (EC) signals can be alternatively obtained through DNA-MB or DNA-RuHex systems. MB, methylene blue; MCH, mercaptohexanol; CP, capture probe; SP, signal probe; CS, complementary strand. **(b)** Oligonucleotide sequences used for assembly of DNA traditional sandwich (DNA-TS) and DNA superstructure (DNA-SS).

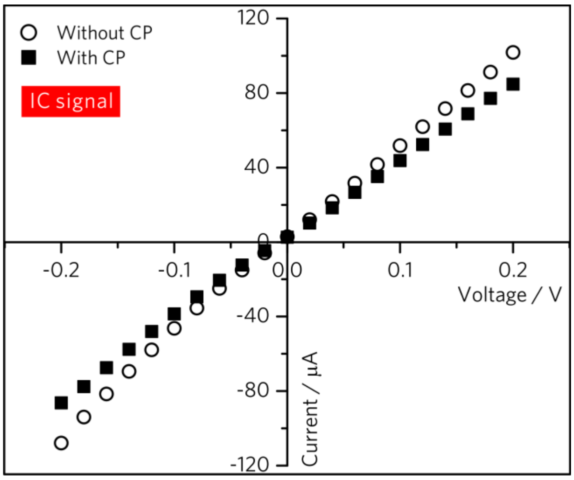

Supplementary Figure 3. *I-V* recordings before (hollow circle) and after (solid square) the capture probe (CP) immobilization at the inner of AAO nanochannels. The concentration of CP was 0.1 μM. Supporting buffer, 500 mM KCl.

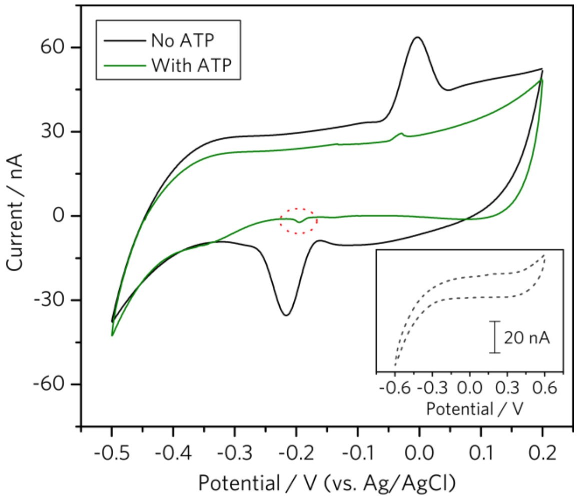

Supplementary Figure 4. Cyclic voltammograms of DNA-TS system tethered with double MB signal reporter before and after treatment with 1 μM ATP. The dotted red circle indicated the sharply decreased electrochemical reduction peak of MB molecules due to ATP mediated disassembly. The concentration of all the probes used for DNA-TS assembly was 0.1 μM. Inset: CV of AAO nanochannels modified by CP and MCH. Supporting electrolyte, 10 mM Tris + 500 mM KCl (pH 7.4); scan rate, 50 mV s^{-1}.

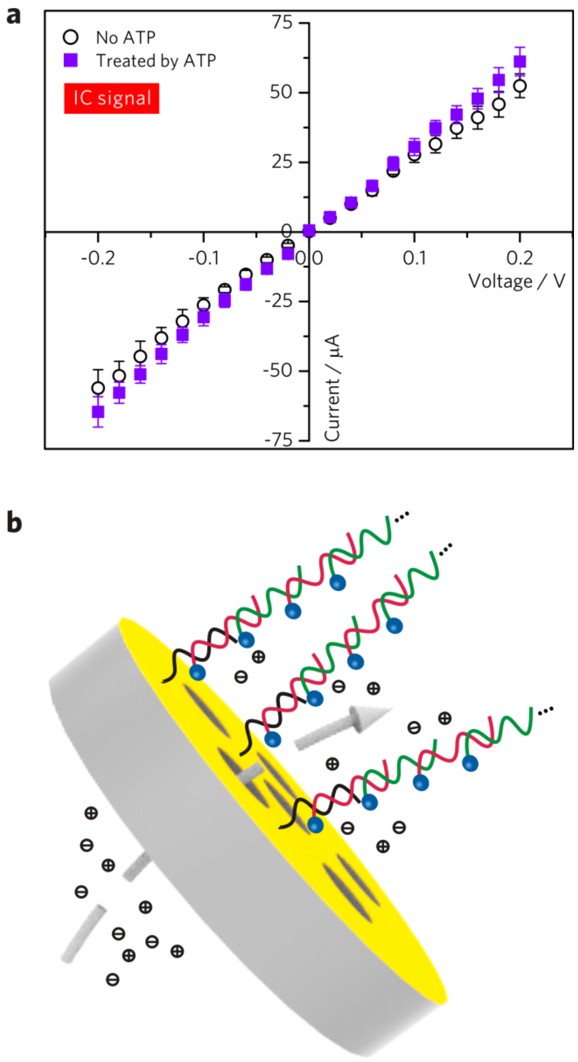

Supplementary Figure 5. (a) *I-V* curves obtained from the assembly of double labeled DNA superstructure (DNA-SS) and the disassembly by ATP at 20-30 nm diameter AAO nanochannels. The slightly changed ionic current (IC) signal suggested that the outer-surface assembled DNA superstructure (here refering to NOSFE) also exhibited negligible ion gating effect. The error bars represented standard deviations from independent three measurements. **(b)** Cartoon describing ions transport through DNA-SS architecture assembled at nanochannels' outer surface.

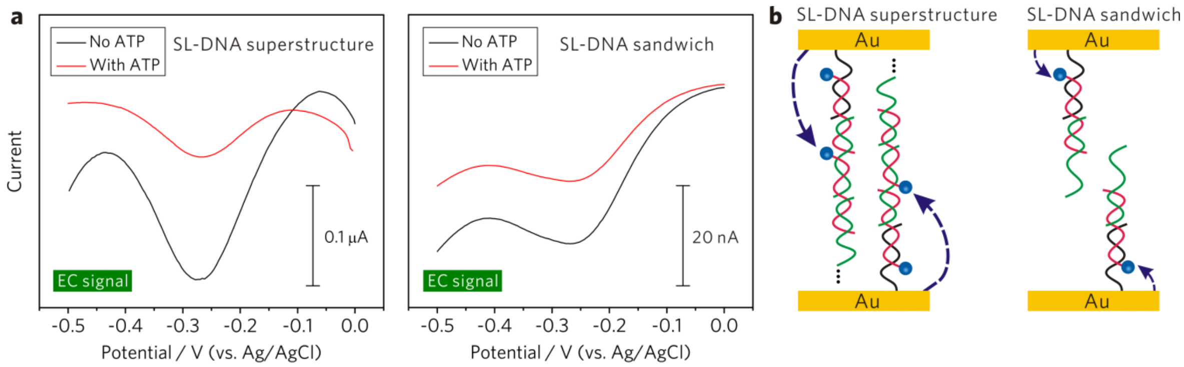

c

DNA architectures	DL-DNA superstructure	SL-DNA superstructure	DL-DNA sandwich	SL-DNA sandwich
Molecular switch efficiency	HIGH	MODERATE	MODERATE	LOW

Supplementary Figure 6. (a) SWV curves recorded at the assembly of SL-DNA superstructure and SL-DNA sandwich, and the disassembly by ATP at the inner wall of nanochannels. SL, single labeling; DL, double labeling. Note that SL represented that only SP1 was tethered with MB tag but SP2 was not. **(b)** Schematic illustration of electron transfer (ET) through the confined DNA architectures. The blue dashed arrows indicated electron transfer (ET) between the MB molecules with the gold surface. **(c)** Summary of electrochemical molecular switch efficiency of different DNA-MB configurations. Here, the switch efficiency is divided to three levels according to the SWV signal change originating from ATP-related biorecognition events: 1-50%, LOW; 50-75%, MODERATE; 75-100%, HIGH.

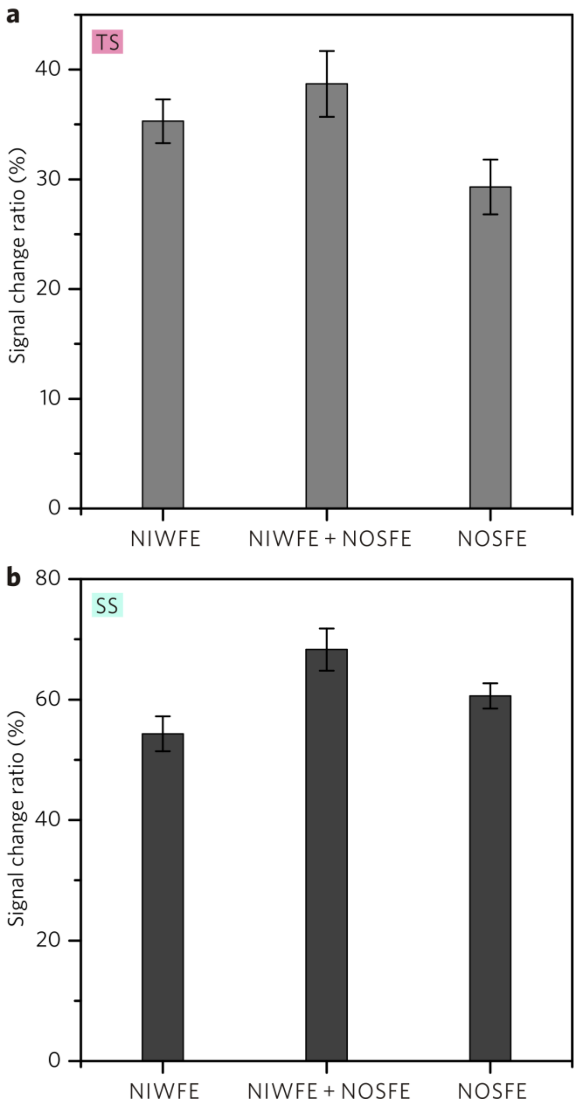

Supplementary Figure 7. Bar graphs showing EC signals change ratios upon the binding of ATP through (a) DNA traditional sandwich (TS) and (b) DNA superstructure (SS) based functional elements using single labeling strategy at nanochannels.

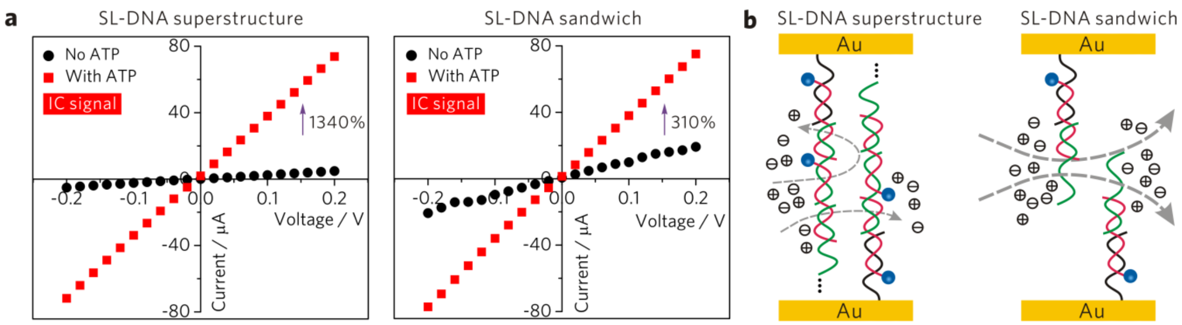

c

DNA architectures	DL-DNA superstructure	SL-DNA superstructure	DL-DNA sandwich	SL-DNA sandwich
Ion gating efficiency	STRONG	STRONG	WEAK	WEAK

Supplementary Figure 8. (a) *I-V* curves recorded at the assembly (circles) of SL-DNA superstructure and SL-DNA sandwich at the inner wall of nanochannels and the disassembly (squares) by ATP. **(b)** Schematic illustration of ions transport (IT) across the confined DNA architectures. **(c)** Summary of ion gating efficiency of different labeling strategy (SL and DL) and DNA architectures (TS and SS). SL, single labeling; DL, double labeling.

Gating Efficiency (%)			
Gating systems	NOSFE	NIWFE	NIWFE + NOSFE
Low-efficency gating	12.6%	217%	248%
High-efficency gating	9.4%	1356%	2285%

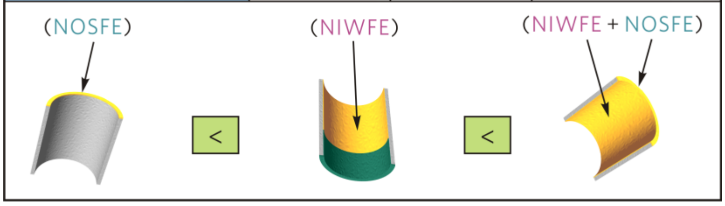

Supplementary Figure 9. Comparison of ion gating efficiency of nanochannels' regional functional elements (FE) constructed by DNA traditional sandwich (TS, low-efficiency gating systems) and DNA superstructure (SS, high-efficiency gating systems).

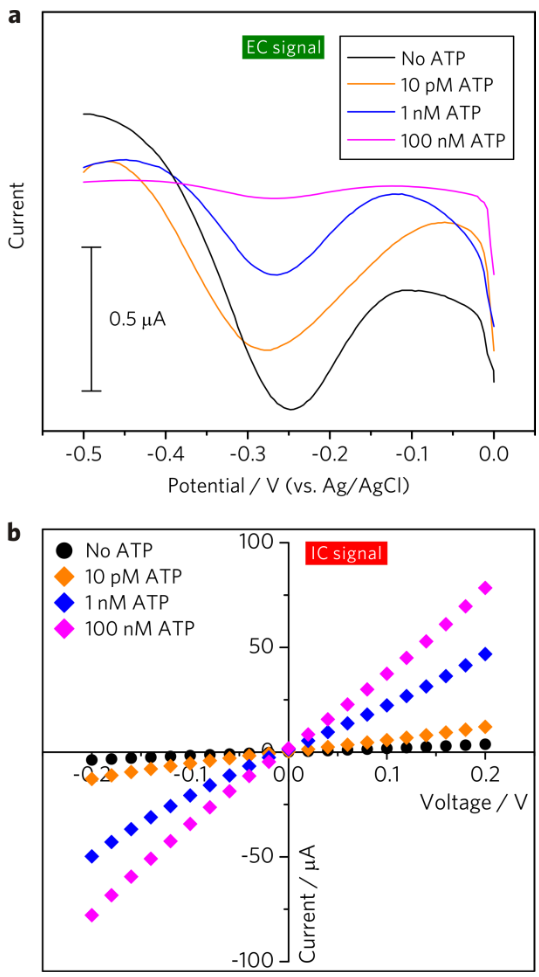

Supplementary Figure 10. Dose-dependent electrolytic current (a) and ionic current (b) response. Shown here were the representative signal profiles through nanochannels inner assembly of double-MB labeled DNA SS and the disassembly triggered by serial concentrations of ATP. Supporting electrolyte, 500 mM KCl.

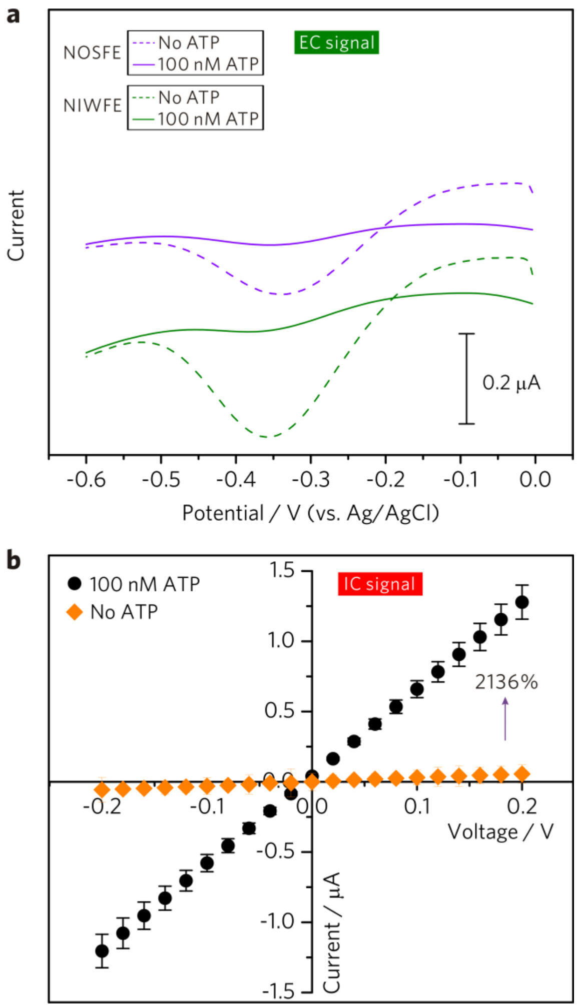

Supplementary Figure 11. (a) Typical DPV curves recorded at the assembly of DNA SS-RuHex system and the disassembly by ATP at the outer surface (NOSFE) and the inner wall (NIWFE) of nanochannels; respectively. **(b)** *I-V* curves through nanochannels inner wall assembled DNA SS-RuHex system and the disassembly by ATP. Supporting buffer, 10 mM Tris (pH 8.0).

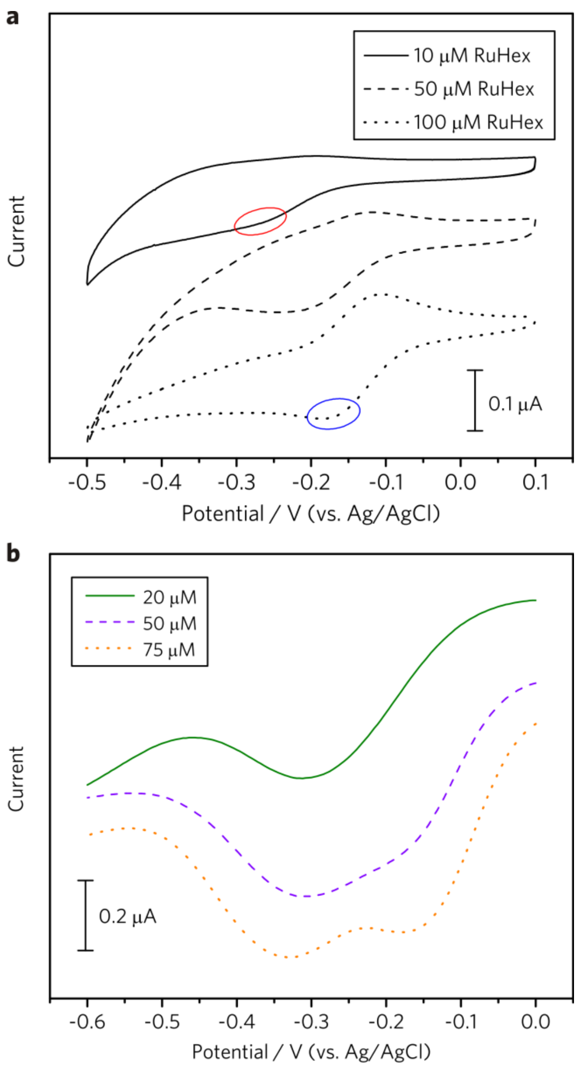

Supplementary Figure 12. (a) Electrochemical behaviors of redox probe RuHex ions entrapped at DNA superstructure (DNA-SS) inner the nanochannels. The red and blue circles indicated the characteristic adsorption peak and diffusion peak of RuHex ions; respectively. Scan rate, 50 mV s^{-1}. **(b)** DPV profiles of different concentration of RuHex ions adsrobed onto DNA-SS framework. Obviously, with the increase of RuHex ions concentration (e.g., > 50 μM) the diffusion peak at -0.15 V tended to appear. Supporting electrolyte, 10 mM Tris (pH 8.0).

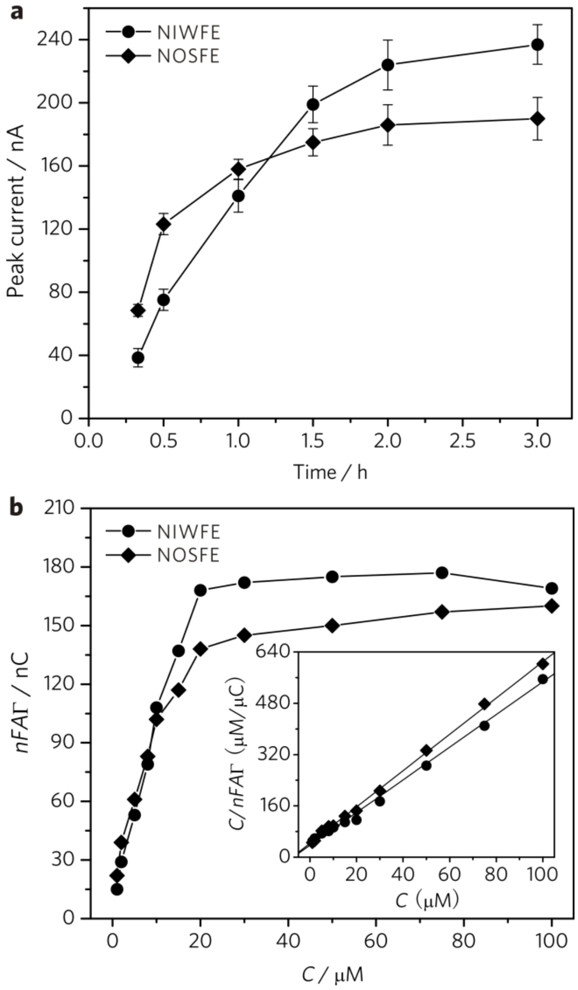

Supplementary Figure 13. (a) Adsorption time-dependence of DPV response of the DNA SS-RuHex system regionally assembled at the inner wall (NIWFE, circle) and outer surface (NOSFE, diamond) of the nanochannels. **(b)** Binding isotherm for RuHex ions on the DNA SS architecture assembled at the inner wall (NIWFE, circle) and outer surface (NOSFE, diamond) of the nanochannels. The inset was a fit of the binding data to Langmuir adsorption.

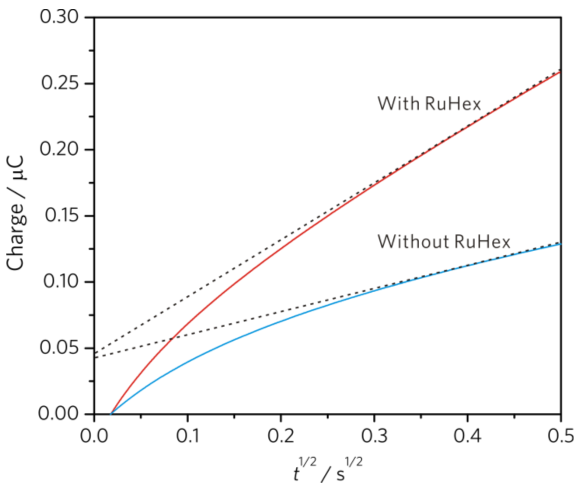

Supplementary Figure 14. Chronocoulometric curves of MCH covered gold surface in the nanochannels with or without 10 μM RuHex treatment. The intercepts shown in dotted lines were nearly equivalent whether RuHex was used or not, suggesting negligible nonspecific adsorption of RuHex on the MCH monolayer.

Supplementary Note 1.

Calculation of probe density.

The probe denstity can be calculated based on the following equation:

$$Q = \frac{2nFAD_0^{1/2}C_0}{\pi^{1/2}} t^{1/2} + Q_{dl} + nFA\Gamma_0$$

where n is electron transfer number, F is the Faraday constant, A is the electrode area (cm^2), D_o is the diffusion coefficient (cm^2 s^{-1}), C_o is the bulk concentration (mol cm^{-2}), Q_{dl} is the capacitive charge (C), and $nFA\Gamma_o$ is the charge from the reduction of Γ_o (mol cm^{-2}) of adsorbed redox marker.

$$\Gamma_{DNA} = \Gamma_0 (Z/m) N_A$$

where Γ_{DNA} is the probe surface density (molecules cm^{-2}), m is the number of bases in the probe DNA, Z is the charge of the redox molecule, and N_A is Avogadro's number.

Supplementary References

1. Liu, J. S. et al. Significant enhancement of the adhesion between metal films and polymer substrates by UV-ozone surface modification in nanoscale. *ACS Appl. Mater. Interfaces* **8**, 30576-30582 (2016).
2. Xia, F. et al. An electrochemical supersandwich assay for sensitive and selective DNA detection in complex matrices. *J. Am. Chem. Soc.* **132**, 14346-14348 (2010).
3. Yang, F. et al. Template-independent, in situ grown DNA nanotail enabling label-free femtomolar chronocoulometric detection of nucleic acids. *Anal. Chem.* **86**, 11905-11912 (2014).
4. Shen, L. et al. Electrochemical DNAzyme sensor for lead based on amplification of DNA-Au bio-bar codes. *Anal. Chem.* **80**, 6323-6328 (2008).
5. Steel, A. B., Herne, T. M. & Tarlov, M. J. Electrochemical quantitation of DNA immobilized on gold. *Anal. Chem.* **70**, 4670-4677 (1998).
